# Epidemiological findings of childhood ocular trauma in a public hospital in Colombia

**DOI:** 10.1186/s12886-021-02014-1

**Published:** 2021-06-05

**Authors:** Valeria D’Antone, Diana Cristina Palencia Flórez, Claudia Juliana Lopez García, Flor De María Cáceres Manrique, Nahyr López Barbosa

**Affiliations:** 1 Specialized in Anterior Segment, Optometry School, Saint Thomas University, Bucaramanga, Colombia; 2 Saint Thomas University, Bucaramanga, Health division, Faculty of Optometry, Cra 27 No 180-395, Bucaramanga, Colombia; 3 Saint Thomas University Bucaramanga, Bucaramanga, Colombia; 4 Public Health Department, Universidad Industrial de Santander, Bucaramanga, Colombia

**Keywords:** Eye injuries, Closed globe injury, Open globe injury, Visual acuity, Child

## Abstract

**Background:**

Eye injury is a serious worldwide public health problem that may cause blindness. In children, blindness has functional impact and psychosocial implications. As indicated in many worldwide studies, identification of risk factors associated with the socio-cultural context may prevent eye injuries. The objetive of the study is to describe the sociodemographic and epidemiological characteristics of pediatric eye injury and its effects on ocular structures in a public hospital from Colombia.

**Method:**

A retrospective cross-sectional study was carried out between January 1, 2015, and December 31, 2017, in a tertiary public hospital of a medium-sized city located in the Northeast of Colombia. Children under 15 years old with trauma to the eyeball or its adnexa were included. The Birmingham Eye Trauma Terminology System (BETTS) was used. Eye burns and Ocular adnexa were also included.

**Results:**

61 cases of eye injuries were recorded, 67.21% (41 cases) of which were males. 57.37% (35 cases) corresponded to closed-globe injuries both contusion and lamellar laceration. Visual acuity fluctuated between 20/20 and 20/40. 14.75% (9 cases) were open-globe injuries while 50% (4 cases) were penetrating trauma. 27.86% of the injuries (17 cases) did not directly compromise the eyeball, 58.82% (10 of these cases) of which corresponded to eyelid wounds, and neither of those had visual acuity information.

**Conclusion:**

The study showed that the majority of eye injuries in children under 15 years old, from a public hospital in the Northeast of Colombia, are closed globe, caused by blows, and occur in males.

## Introduction

Eye trauma or eye injury (EI), as indexed by the Medical Subject Headings (MeSH), is a serious public health problem involving psychosocial implications and can be prevented in 90% of cases [1–3]. Globally, 1.6 million people develop blindness as reported by Negrel et al. in 1998 [4]. Every year, serious ocular trauma affects a quarter of a million children [5]. According to Scruggs et al., in the United States between 2003 and 2007, EI was considered the main cause of unilateral blindness affecting 40,000 to 60,000 patients annually [6].

For the year 2000, 2.4 million eye injuries were estimated per year; 35% of which occurred in people 17 years old or younger according to Brophy et al. [7]. Most childhood EI occur in recreational environments and are caused by physical mechanisms such as toys or artifacts that can be easily manipulated by children. Adult traumas, however, usually occur due to occupational accidents [8–11].

Using extrapolated data from the global population, it is estimated that between 160.000 and 280.000 children under 15 suffer severe EI every year and most require hospitalization [5]. Studies show that males are affected more than females with a ratio ranging from 2: 1 to 4: 1 [12, 13]. In a study carried out in Santander, Colombia in 2003, the highest percentage of children with ocular trauma was between 0 and 5 years [14]. Blunt and sharp objects were reported as the most common trauma mechanisms. Children were usually alone when the trauma occurred [5].

Given the characteristics and implications of EI, classification systems have been created to standardize diagnoses of professionals worldwide. The Birmingham Eye Trauma Terminology System (BETTS), used in this study, classifies trauma according to injuries to the eyeball walls. These include closed-globe injury (CGI), all those where there is a partial thickness wound, and open globe injury (OGI) in which there is a full-thickness wound on the walls of the globe [15].

In Colombia, few reports are available in this regard, except for a study carried out in a hospital in the northeast in 2003. Therefore, this research describes the sociodemographic and epidemiological characteristics of pediatric eye injury in the only public hospital in Bucaramanga, Colombia.

This characterization contributes towards exploring medical care in a tertiary public hospital and helps to define the potential risk factors associated with pediatric eye injury in a medium-sized city in a Latin America Country.

## Methodology

A retrospective cross-sectional study was carried out between January 1, 2015 and December 31, 2017 with the participation of the University Hospital of Santander (HUS by its acronym in Spanish), which facilitated the collection of information.

### Eligibility criteria

Children under 15 years of age with trauma to the eyeball or its adnexa were included. Clinical reports that had little clinical correlation of data or erroneous diagnoses were excluded.

### Setting and sample

Through a non-probabilistic sampling for convenience, eye injuries in children over a three year peiod in the Ophthalmology Services of the University Hospital of Santander and who met the selection criteria were included.

### Clinical evaluation

Initially, patients were evaluated in the emergency room of the HUS, and in the case of alterations affecting the eyes, they were referred to the Ophthalmology Unit where they were evaluated by optometrists and ophthalmologists.

Demographic information, date of injury (cause, mechanism, type, clinical signs) and visual outcome were recorded for medical records. The ocular examination was carried out with a direct ophthalmoscope, slit lamp, and an indirect ophthalmoscope was used to explore the fundus in case of clear ocular media with a + 20 Diopter lens. Visual acuity was mesured using the Landolt C chart and Snellen’s charts.

The information available in the medical record was reorganized according to Birmingham Eye Trauma Terminology. Two oher categories were included: Eye burns and Ocular adnexa injuries.

### Statistical analysis

A univariate descriptive analysis was carried out applying the relevant statistical tests according to the nature and scale of measurement of the variables. Measures of central tendency and dispersion were considered in the case of quantitative variables, while for qualitative variables, proportions calculation was considered.

The normality of the continuous data was tested, and the median was used with the interquartile range (IQR) when a non-normal distribution was presented.

Additionally, bivariate analysis was performed to establish the association between sociodemographic variables (age, sex, education, affiliation to the health system, place of residence) and clinical characteristics such as the type of trauma and its corresponding mechanisms using the Fisher’s Exact Test.

The analysis was done in the software Stata 14.

### Ethical considerations

This paper follows the foundations outlined in the Declaration of Helsinki.

The Ethics Committees in scientific research of the Universidad Industrial de Santander (Colombia) waive the requirement for informed consent to develop the study because the investigation achieves the following:

1. The study involves minimal to no risk to subjects as the only known risk to patients is the possible loss of confidentiality, which has been guarded against by limited personnel access to the database and password protection as well.
2. The waiver does not adversely affect the rights and welfare of the subjects because the study is non-interventional and does not affect the subject’s rights for patient care and does not interfere with welfare. Subject confidentiality is protected by the assignment of a code for identification in the study.
3. The research could not practicably be carried out without the waiver because pediatric ocular trauma is a rare event. For this reason the study had to be carried out retrospectively.
4. The study is non-interventional and providing information to patients is not likely. Also, they are reviewing medical records but are not recording identifiers. They would not be able to link subjects back to the study and therefore would not be able to provide additional information.

Information from the medical records was extracted by two senior Optometry students using a collection format designed for the study.

## Results

**Fig. 1:**
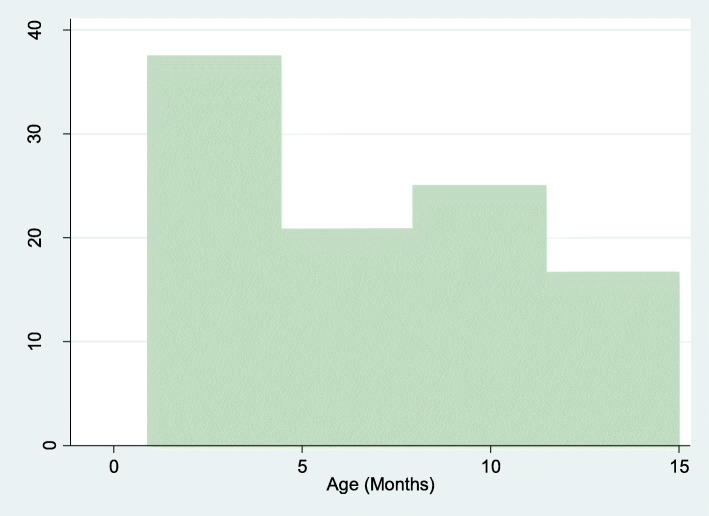
Distribution of eye injury by age

61 pediatric patients, admitted to the University Hospital of Santander from January 1, 2015 to December 31, 2017, were included. Four records were excluded during the selection process because of incomplete medical records. During the period of evaluation, 34.43% (21) of the injuries were registered in March, September and October and 11 year old children had the higest frecuency of trauma 11.47% (7) (See Fig. 1).

67.21% (41 cases) were male. 81.91% (50 cases) resided in urban areas and the median age was 9 (0.91–15). 93.44% of the participants (57 cases) were affiliated with the subsidized health regime. 67.21% (41 cases) were in school and 85.25% (52 cases) came from the department (administrative district in Colombia) of Santander. There were no statistically significant differences by sex in these variables (See Table 1).

**Table 1:** Distribution of sociodemographic characteristics by sex

SOCIODEMOGRAPHIC	TOTAL	MALES	FEMALES	*P* VALUE
CHARACTERISTICS%(n)		*n* = 41	*n* = 20	
AGE (years)	9 (6)^a^	7 (7)	9.41 (5.46)^a^	
HEALTH SYSTEM AFFILIATION				0.5^b^
Contributive	1.64 (1)	2.44 (1)	0 (0)	
Subsidized	93.44 (57)	95.12 (39)	90 (18)	
Personal medical payments	4.92 (3)	2.44 (1)	10 (2)	
SCHOOL				0.5^b^
Yes	67.21 (41)	63.41 (26)	75 (15)	
No	13.11 (8)	17.07 (7)	5 (1)	
Non-report	19.67 (12)	19.51 (8)	20 (4)	
PLACE OF RESIDENCE				0.09^b^
Urban	81.97 (50)	87.8 (36)	70 (14)	
Rural	18.03 (11)	12.2 (5)	30 (6)	
DEPARTMENT^c^				0.56^b^
Arauca	3.28 (2)	4.88 (2)	0 (0)	
Bolívar	6.56 (4)	7.32 (3)	5 (1)	
Cesar	1.64 (1)	0 (0)	5 (1)	
Norte of Santander	3.28 (2)	4.88 (2)	0 (0)	
Santander	85.25 (52)	82.93 (34)	90 (18)	

^a^ Median Interquartile Range ^b^ *P*-value obtained with Fisher’s Exact Test c

Administrative district in Colombia

**Fig. 2:**
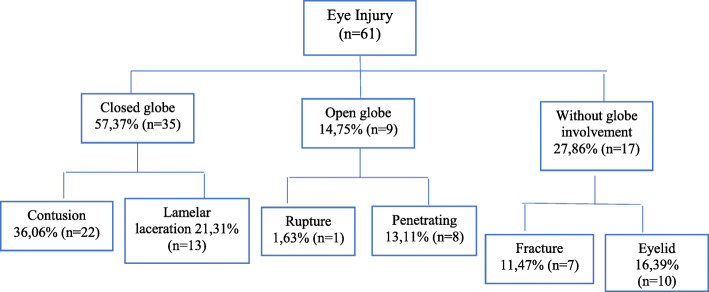
Distribution of the types of trauma according to the BETT classification. Source: The authors

The highest prevalence found corresponded to mixed EI (75.41% (46 cases)) considered as injuries that affect more than one ocular structure at the same time. Of the total of 61 patients, 35 involved closed-globe injuries, 9 were OGI and 17 were without globe involvement (see Fig. 2).

There was a higher prevalence of closed lamellar laceration trauma with 53.85% (7 cases) with visual acuity between 20/20 and 20/40, followed by 50% (4 cases) for penetrating OGI. No statistically significant relationships were evidenced (See Table 2).

**Table 2:** Initial visual acuity by type of trauma

Visual acuity	Closed globe		P value^**b**^	Open globe		P value^**b**^	Without globe involvement		P value^**b**^
	Contusion	Lamellar laceration		Rupture	Penetrating		Fracture	Eyelid	
**20/20 –**	45.45 (10)	53.85 (7)	0.24	0(0)	50 (4)	0.55	14,29 (1)	30 (3)	0.56
**20/40**									
**20/50 –**	13.64 (3)	7.69 (1)		0 (0)	12.50 (1)		14,29 (1)	0 (0)	
**20/100**									
**20/200 –**	0 (0)	15.38 (2)		0 (0)	25 (2)		0 (0)	0 (0)	
**LP**^**a**^									
**Non- report**	40.91 (9)	23.08 (3)		100 (1)	12.50 (1)		71.43 (5)	70 (7)	

^a^ Light perception ^b^ *P* value obtained by Fisher’s exact test

When analyzing EI occurrence, the highest proportion happened in the street with 32.79% (20 participants) followed by 26.23% (16 cases) occurrimg at home (See Table 3).

**Table 3:** Distribution of the types of trauma and place of occurrence by age

	0.91–5 years	6–10 years	11–15 years	P Value^**a**^
	n = 20	*n* = 21	n = 20	
**Type of trauma %(n)**				
**Closed Globe (*n*** **= 35)**				
Contusion	22.73 (5)	40.91 (9)	36.36 (8)	0.73
Lamellar laceration	15.38(2)	53.85 (7)	30.77 (4)	
**Open Globe (*n*** **= 9)**				0.44
Rupture	0 (0)	0 (0)	100 (1)	
Penetrating	0 (0)	62.5 (5)	37.5 (3)	
**Without Globe involvement (*n*** **= 17)**				0.83
Fracture	57.14 (4)	14.29 (1)	28.57 (2)	
Eyelid	40 (4)	20 (2)	40 (4)	
**Place % (n)**				
Home	40.0 (6)	20.33 (5)	22.73 (5)	
School	0 (0)	0 (0)	9.09 (2)	
Street	20 (3)	29.17 (7)	45.45(10)	
Others	6.67 (1)	29.17 (7)	13.64 (3)	0.17
Non-refer	33.33 (5)	20.83 (5)	9.09 (2)	

^a^*P* value obtained by Fisher’s exact test

Likewise, 45.71% (16) of closed-globe injuries, the most frequent of which were contusions, occurred in minors between 6 to 10 years of age (56.25% = 9 children). While open globe injuries 55.56% (9), corresponding to penetrating injuries, also occurred in minors between 6 to 10 years of age. Fractures and eyelid injuries without globe involvement 47.06% (8) registering the same frequency, occurred in children under five years of age. No statistically significant differences are evidenced when analyzing types of trauma by age group (See Table 3).

### Evaluation of ocular structures

Conjunctival hyperemia 47.54% (29 cases) were found to be the most prevalent clinical characteristics, followed by 26.23% (16 cases) of eyelid edema and corneal laceration 11.47% (7 cases). Additionally, less than 5% of the children presented non-reactive pupil, hyphema, scleral erythema or palpebral ecchymosis, among others signs. 70.49% of the total population (43 cases) required hospitalization and medication for blunt-type eye trauma that occurred in 62.86% (12 of the cases). Pharmacological management was used in 86.36% (19 cases).

Lamellar lacerations, 37.14% (13 cases), 76.92% (10 cases) required medication management. There was no statistically significant association between choice of treatment and the type of trauma.

**Fig. 3:**
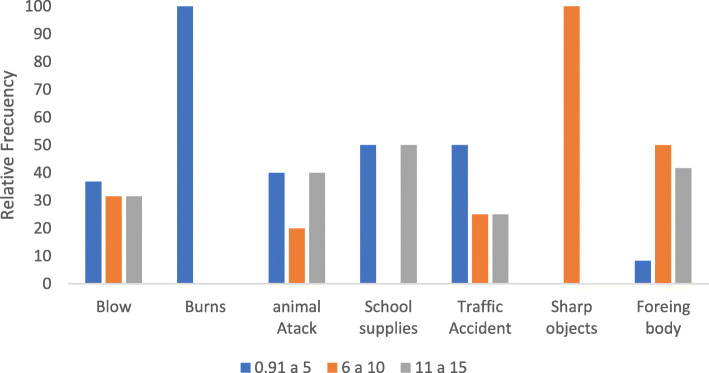
Distribution of the trauma mechanism by age group. Source: The authors

The most frequent EI mechanism in all age groups (63.93% (39)) were blows, followed by foreign body injuries, representing 19.67% (12), the second most frequent in children between 6 to 15 years of age (See Fig. 3).

## Discussion

Eye injuries are one of the major causes of morbidity and blindness in the pediatric population [16–18]. In Colombia, little data is available in this regard, except for a study carried out by Serrano et al. between January 1996 and December 2000 [14] and 7 cases reported by Tello et al. in another hospital, in the same city between 2013 and 2018. The same team carried out another study in 2014–2015 but in an adult population.

In the present study, 61 childhood EI were recorded. The highest percentage 67.21% (41 cases) were male, which is consistent with worldwide studies [19–21]. There was a predominance of OGI in 57.37% (35 of the cases) as was the case in the study by Serrano et al. in which a prevalence of 82.89% was obtained. In contrast, injuries to the ocular adnexa and the orbit presented a prevalence of 27.86% (17 cases) of childhood EI. This finding is also recorded in the study of Gise et al. in the United States with a prevalence of 39.1% of lesions in ocular adnexa and 35.8% of orbital traumas in the period 2008–2014 [22].

The greatest number of injuries occurred in the 6 to 10-year-old group with 39.34% (24 of the cases), followed by the 11 to 15-year-old group - 36.06% (22 cases). These data are similar to those reported by Prakash et al. in India for 2017, where a higher percentage of trauma was found in children between 11 and 15 years old [23]. The above is also related to data recorded by Huda et al. in the 2005–2009 period. In this study, there was a higher prevalence in the 6 to 14-year-old group [24]. In Kadappu et al. in Australia, there was a higher proportion of trauma among the 9 to 14-year-olds for the period 2000–2008 [25].

Blows to the eyes represented 86.67% of the cases, with 19.67% being the result of falls. This differs from several studies such as that of Ricardo Marti et al. in Cuba, who show the prevalence of blows by spinning tops in 24.3% of the population in 2015 [26]. Ebrahim et al. in Egypt report that 20% were due to trauma caused by broomsticks for the year 2016 [18], and Singh et al. in India show that by 2017, 29.54% of the injuries were caused by organic objects such as tree branches and sticks [27]. In contrast, in the United Kingdom, according to Abbott et al., childhood EI was caused by compressed air guns in 53% of the cases [5]. This implies that the mechanisms are related to the presence of objects that are within the reach of children for their recreational activities.

32.79% (20 cases) happened outdoors. This data contradicts those reported by Serrano, where the home was found to be the place of greatest occurrence with 44.4% of the cases [14], as was also the case in the study by Huda et al. with 42.5% occurring at home [24]

A value between 20/20 and 20/40 visual acuity was recorded for lamellar laceration CGI. This VA data was also recorded by Archambault et al. in Canada between 2007 and 2010 [28] for contusion-type CGI and by Serrano et al. in Northeast Colombia [14] with a minimal difference in the VA range between 20/20 and 20/50.

Finally, during the development of the present study, flaws were identified in the registry of visual acuity in 42.62% of the clinical histories and non-standardization in the description of the alterations by structure. In the same way, according to the characteristics of the study population, the inference of the results could be limited to children under 15 years old, residents in urban areas, schooled, with subsidized affiliation to the health system and that receive medical care at the public hospital. As limitations, we recognize the sample size and the lack of information related to the evolution of the cases.

## Conclusions and recommendations

The study showed that childhood eye traumas in the Northeast Colombian region, are more frequent in males. By age group, it presents a higher percentage of blunt globe injuries between 6 and 10-year-old children. Regarding ocular structures, injury is recorded in one or more of them, being classified as a mixed commitment.

Considering that falls represented a frequent mechanism of pediatric EI, it could be inferred that having permanent supervision of parents or responsible adults in daily activities and entertainment would help reduce the number of cases.

Difficulty in classifying some eye injuries by BETT brings some trouble to our study because most of the EI were found in the adnexa structures. For future investigations, all eye injuries, not just the globe in the classification system, should be added.

In the medical records, it is important that visual acuity be reported and that the description of semiology terms be standardized in order to streamline the evolution of the patients and to facilitate the investigation process.

## Data Availability

Due to the nature of this research, the hospital that allowed access to information did not agree for their data to be shared publicly. Supporting data are only available in the case of special requirement. Contact diana.palencia@ustabuca.edu.co
